# School-aged children’s movement behaviours and subjective health complaints in Japan: a cross-sectional study during COVID-19 pandemic-related school closures and after school reopenings

**DOI:** 10.1186/s12889-024-18712-6

**Published:** 2024-04-30

**Authors:** Akane Kasai, Akiko Shikano, Ryo Tanaka, Mari Yoshinaga, Shingo Noi

**Affiliations:** 1 Graduate School of Health and Sport Science, Nippon Sport Science University, Tokyo, Japan; 2 Research Institute for Children’s Physical Health, Nippon Sport Science University, Tokyo, Japan; 3 School of Sport Sciences, Osaka University of Health and Sport Sciences, Osaka, Japan; 4 Faculty of Pharmaceutical Science, Showa Pharmaceutical University, Tokyo, Japan

**Keywords:** Physical activity, 24-hour movement behaviour guidelines, Screen time, Sleep, Mental health, Psychosomatic symptoms

## Abstract

**Background:**

Social restrictions associated with the COVID-19 pandemic have altered children’s movement behaviours and impacted their mental health. However, the influence of social restrictions on subjective health complaints remains inadequately understood. This study compared adherence to 24-hour movement behaviour guidelines and the prevalence of subjective health complaints during school closure and one year after reopening. We also examined how combinations of adherence to movement behaviour recommendations relate to subjective health complaints.

**Methods:**

A repeated cross-sectional survey was conducted at two points. The first survey in May 2020 included 1535 (766 boys and 769 girls) participants during school closures, while the second survey from May to July 2021 involved 1125 (583 boys and 542 girls) participants one year after school reopening. The questionnaire covered socio-demographics, physical activity, screen time, sleep, and subjective health complaints. Differences between periods were analysed using chi-square tests. Logistic regression models assessed the association between adherence to guidelines and subjective health complaints.

**Results:**

During school closure, children were more likely to meet ‘only sleep’ recommendations and have irritability and lethargy symptoms. Irrespective of sex, those adhering to two or all three recommendations (excluding physical activity and screen time) had a lower risk of symptoms related to physical and mental pain, fatigue, irritability, and lethargy as compared to those who met none of the recommendations.

**Conclusions:**

Children should meet at least one physical activity or screen time recommendation in addition to sleep recommendations for subjective health. Strategies considering the priority of each movement behaviour are crucial, even during abnormal situations, such as pandemic-related social restrictions. This study offers insightful findings concerning children’s mental health issues during unprecedented and massive disasters or crises.

## Background

The emergence of the coronavirus disease (COVID-19) in December 2019 has profoundly impacted children’s daily lives globally, with various restrictions including social distancing and quarantine [1, 2]. Studies have indicated notable shifts in children’s movement behaviour during pandemic-associated restrictions, including: reduced physical activity [3–5]; increased sedentary behaviour, particularly recreational screen time [3–6]; and heightened sleep duration [5–7]. Additionally, several international studies have suggested that changes in movement behaviours and pandemic-induced social distancing measures affect physical health and have implications for children’s mental health, including depression [8–10], anxiety [8, 9, 11], well-being [12], and mood [13].

While substantial research on the association between movement behaviours and health outcomes exists, most studies have examined individual aspects, such as hours of physical activity, screen time, and sleep, often with only partial adjustment for other movement behaviours [14]. Typically, these studies consider the health benefits of each movement behaviour separately. In recent years, ample evidence has emphasised the significance of interactions among movement behaviours. The interest of these studies lies in understanding how movement behaviours, such as physical activity, sedentary behaviour, and sleep, spanning the entire 24-hour period, affect each other and contribute to children’s comprehensive overall health. Canada developed the first integrated 24-hour movement behaviour guidelines for school-aged children and youth in 2016 [15]. These guidelines recommend that children aged 5–13 engage in at least 60 min of moderate-to-vigorous physical activity daily, spend no more than two hours of recreational screen time per day, and sleep 9–11 h per night. In Japan, the number of studies reporting the association between the 24-hour movement behaviour guideline recommendations and health outcomes among children is limited [16–18]. Therefore, further research is required to examine the relationship between movement behaviours and various health indicators, determining the applicability of the existing 24-hour movement behaviour guidelines for Japanese children.

Even before the pandemic, poor mental health in children was a global public health concern, with subjective health complaints presenting a severe problem in many countries [19–21]. Subjective health complaints encompass symptoms experienced by individuals regardless of the presence of a specific diagnosis. These symptoms include abdominal pain, headache, backache, irritability, lack of concentration, and diverse stress-related problems [22]. Subjective health complaints can serve as a target for interventions aimed at promoting health and preventing future mental health problems, including psychological disorders [23]. Additionally, subjective health complaints could have been exacerbated among children during the pandemic. Investigations in Germany revealed a decrease in health-related quality of life and a considerable increase in mental and psychosomatic health complaints among children and adolescents from the pre-pandemic to the pandemic period [1, 24]. However, the differences in subjective health complaints between abnormal situations, such as social restrictions due to the pandemic, and ordinary situations among children are not yet adequately understood.

Additionally, the association between adherence to the 24-hour movement behaviour guideline recommendations and subjective health complaints among children has not yet been investigated. Though several studies have examined the association between movement behaviours and subjective health complaints, they have not utilized the 24-hour movement behaviour guidelines. An examination in Ireland reported that both physical activity and screen time were associated with the risk of subjective somatic and psychological health complaints [19]. Similar results have been observed in surveys conducted in several European and North American countries [25]. Moreover, a study in Spain indicated that sleeping for over 10 h was significantly associated with reporting no health complaints [26]. These studies indicate a positive impact of healthy movement behaviours on children’s subjective health. Prior findings have examined the relationships between the 24-hour movement behaviour guideline recommendations and mental health indicators, including depression [27, 29–31], anxiety [27, 29–31], internalizing and externalizing behaviours [32], health related quality of life [28, 33], and self-rated health [34] among children and adolescents. Therefore, it is expected that 24-hour movement behaviours will be associated with subjective health complaints.

Hence, this study compared the adherence to 24-hour movement behaviour guidelines and the prevalence of subjective health complaints during school closure and one year after school reopening. We hypothesised that during school closure, there will be a decline in adherence to movement behaviour recommendations and an increase in subjective health complaints. Additionally, this study examined the association between meeting different movement behaviour recommendations and subjective health complaints. Since meeting various movement behaviour recommendations is associated with good mental health, a higher adherence to these recommendations is likely to be associated with a lower risk of experiencing subjective health complaints.

## Methods

### Study design and participants

We conducted a repeated cross-sectional survey in May 2020 as the first survey during school closure and a second survey from May to July 2021, following the end of the second state of emergency declaration, one year after schools reopened in Japan. Nationwide school closures started in March 2020, following the state of emergency by the Japanese government. and continued until May 2020. One year after the national-level school closure was lifted, children had been living their daily lives with COVID-19 despite experiencing temporary school closures depending on the current infection situation. The Ethics Committee of Nippon Sport Science University approved this study (no. 020-H002).

Using snowball sampling, we enrolled 3108 students from the first to sixth grades (aged 6–12 years) across 22 public elementary schools in Japan. After removing those with missing responses, we analysed 2660 participants’ data: 1535 (766 boys and 769 girls) during closure and 1125 (583 boys and 542 girls) after reopening.

### Procedure

We collected data via an anonymous online questionnaire using Google Forms to reduce the spread of COVID-19. Prior to the survey, we contacted all school heads, asking them to distribute the survey link to students’ parents or legal guardians via emails or documents. These also included an explanation of the study’s purposes and expected benefits. Each participant’s parent or legal guardian was provided information about participants’ research rights (i.e. voluntary participation, independence from school grades in their responses) in the part of the online form description. They were instructed to complete forms with demographic information about their children if they consented to participate. Children answered the questions themselves; however, if it was difficult, they did so with their parents, or their parents answered in their stead. We used a self-administered questionnaire comprising questions about socio-demographics (sex and grade), physical activity, screen time, sleep, and subjective health complaints.

### Measures

#### Physical activity

Physical activity was assessed using the question, ‘How many days in the past seven days did you engage in at least 60 minutes per day of physical activity?’ The validity of this question for Japanese children has been confirmed by a previous study [35]. Furthermore, this question was recommended as a quick surveillance tool [36] and was used in a previous study on physical activity, screen time, and subjective health complaints among school-aged children [19]. Adherence to the physical activity recommendation was defined as participants obtaining at least 60 min of physical activity daily, because the Canadian 24-hour movement behaviour guidelines recommend an accumulation of at least 60 min/day of moderate-to-vigorous physical activity [15].

#### Screen time

Screen time was assessed by measuring the daily use of digital devices for recreation. Recreational screen time included the use digital devices (smartphones, tablets, and PCs), watching television and videos, and playing digital games (TV, computer, and mobile games). Although there is no universally accepted gold standard for measuring screen time, questions similar to those used in the Japanese national survey [37] were applied in this study. Adherence to the screen time recommendation was defined as a total screen time of no more than two hours per day [15].

#### Sleep

We measured bedtime and wake-up time with the questions: ‘What time do you go to bed on weekdays recently?’ and ‘What time do you wake up on weekdays recently?’ that were used in the large-scale national surveys of Japanese children [38, 39]. Sleep duration was calculated from these values. Participants were categorised as meeting the sleep recommendation if they obtained 9–11 h of sleep per night [15].

#### Subjective health complaints

Subjective health complaints were assessed with a 22-item questionnaire including statements such as ‘I have a stomach-ache’, ‘I have a stiff neck’, ‘I am irritated’, and ‘I am not motivated’. This questionnaire was constructed by referring to the ‘Jikaku-sho shirabe’, a questionnaire produced by the Industrial Fatigue Research Committee of Japanese Occupational Health [40]. The reliability and validity of ‘Jikaku-sho shirabe’ in school-aged children have been confirmed [41]. Participants answered the following question: ‘How much do you agree or disagree with each of the following statements about your recent health?’ using four options: ‘strongly agree’, ‘agree’, ‘disagree’, and ‘strongly disagree’.

### Statistical analysis

Participants were classified into the following eight categories depending on their adherence to the 24-hour movement behaviour recommendations: none; only physical activity; only screen time; only sleep; physical activity and screen time; screen time and sleep; physical activity and sleep; or physical activity, screen time, and sleep. We subsequently compared the proportion of participants in each category during school closure and one year after school reopening using chi-square tests. As this analysis involved more than two groups, we calculated the adjusted standardised residuals to identify deviations from the expected frequency and to determine which residuals were smaller or greater than 1.96.

Exploratory factor analysis using the maximum likelihood method was conducted to investigate the factor structures of subjective health complaints. The data of subjective health complaints used for this analysis were obtained from the first and second surveys. Factors with eigenvalues under 1.0 were discarded based on Kaiser’s criterion, and the number of factors was determined. Subsequently, oblique promax rotation (factor loadings (> 0.40) was applied. McDonald’s omega was used to estimate the internal consistency of the scale and was regarded as having good reliability if the coefficient was above 0.70.

Based on the results of the factor analysis, chi-squared tests were performed to compare the proportions of subjective health complaints during school closure and one year after reopening. In analysing proportions of subjective health complaints, the four options mentioned above (‘strongly agree’, ‘agree’, ‘disagree’, and ‘strongly disagree’) were consolidated into two categories, with ‘strongly agree’ and ‘agree’ combined as ‘agree’, and ‘strongly disagree’ and ‘disagree’ merged as ‘disagree’.

Logistic regression models were employed to estimate the association between adherence to 24-hour movement behaviour guidelines and subjective health complaints using the forced entry method. In these models, the dependent variables were subjective health complaints, and the independent variables included socio-demographics (sex and grade), periods, and adherence to movement behaviour recommendations. Subjective health complaints were converted into binary variables based on whether an individual experienced more than one health complaint (coded 1) or none (coded 0) for each health complaint factor. This determination depended on previous studies that suggest subjective health complaints can co-occur rather than manifest as single symptoms [20, 42, 43]. The independent variables were recorded as follows: sex (0 = boys, 1 = girls), grade (0 = 1st, 1 = 2nd, 2 = 3rd, 3 = 4th, 4 = 5th, and 5 = 6th), period (0 = during school closure, 1 = one year after school reopening), and adherence to movement behaviour recommendations (0 = none, 1 = only physical activity, 2 = only screen time, 3 = only sleep, 4 = physical activity and screen time, 5 = screen time and sleep, 6 = physical activity and sleep, and 7 = physical activity, screen time, and sleep). Odds ratios (ORs) and 95% confidence intervals (CIs) were calculated.

All these analyses, excluding logistic regression analysis, were sex-stratified owing to reported sex differences in mental health issues, including somatic and psychological complaints [44] or subjective symptoms of fatigue [45]. Statistical significance was set at *p* < 0.05. The analyses were performed using IBM SPSS Statistics version 27 (IBM Corporation, Armonk, NY, USA).

## Results

Table 1 presents the proportions of adherence to the 24-hour movement behaviour recommendations during school closure and one year after reopening. Both boys and girls were more likely to meet only sleep recommendations during school closure, whereas the proportion of those who met no recommendation, only screen time recommendations, and screen time and sleep recommendations was greater after the school reopened. Moreover, girls were more likely to meet the only physical activity recommendations after the school reopened.

**Table 1:** Chi-squared test on adherence to 24-hour movement behaviour during school closure/one year after school reopening

	Boys, *n* (%)			Girls, *n* (%)		
	School closure	School reopening	*P*	School closure	School reopening	*P*
None	153 (20.0) ^b^	146 (25.0) ^a^	< 0.001	136 (17.7) ^b^	140 (25.8) ^a^	< 0.001
Physical activity	21 (2.7)	21 (3.6)		7 (0.9) ^b^	15 (2.8) ^a^	
Screen time	16 (2.1) ^b^	46 (7.9) ^a^		19 (2.5) ^b^	50 (9.2) ^a^	
Sleep	415 (54.2) ^a^	189 (32.4) ^b^		438 (57.0) ^a^	154 (28.4) ^b^	
Physical activity and screen time	4 (0.5)	8 (1.4)		2 (0.3)	5 (0.9)	
Screen time and sleep	71 (9.3) ^b^	102 (17.5) ^a^		104 (13.5) ^b^	136 (25.1) ^a^	
Physical activity and sleep	56 (7.3)	43 (7.4)		41 (5.3)	24 (4.4)	
All three	30 (3.9)	28 (4.8)		22 (2.9)	18 (3.3)	

All three comprise physical activity, screen time, and sleep. ^a^ Observed frequency was significantly higher than the expected. ^b^ Observed frequency was significantly lower than expected

Exploratory factor analysis discovered factor structures of subjective health complaints in boys and girls. The results for boys and girls are presented in Tables 2 and 3, respectively. In boys, one item (‘I feel heavy’) was excluded because of a factor loading below 0.40. Similarly, for girls, two items (‘I feel heavy’ and ‘My brain is foggy’) were excluded. The factor structures in boys and girls were similar and, therefore, termed simultaneously. The first factor, encompassing nine items for both sexes, was ‘physical and mental pain’. The second factor, termed ‘fatigue’, comprised six factors in boys and five factors in girls. The third and fourth factors, comprising three items regardless of sex, were named ‘irritability’ and ‘lethargy’, respectively. All McDonald’s omega coefficients met the criteria, confirming the acceptability of the selected factors.

**Table 2:** Factor analysis on subjective health complaints in boys

	Factor loading			
	Factor 1	Factor 2	Factor 3	Factor 4
***Physical and mental pain*** *(ω = 0.912)*				
I have a stomach-ache	**0.897**	-0.014	-0.041	-0.061
I have abdominal pain	**0.804**	-0.127	-0.020	0.059
I have diarrhoea	**0.792**	-0.018	-0.058	0.014
I have heart palpitations	**0.772**	-0.114	0.073	-0.028
I feel nauseous	**0.680**	0.051	-0.112	-0.005
I feel dizzy	**0.665**	0.238	-0.072	-0.053
I have a headache	**0.660**	0.096	0.020	0.013
I feel depressed	**0.598**	0.066	0.125	0.063
I feel like crying	**0.579**	0.001	0.218	0.033
***Fatigue*** *(ω = 0.851)*				
I have a stiff neck	-0.038	**0.837**	-0.007	-0.093
I have eyestrain	-0.066	**0.656**	0.026	0.071
I have a backache	0.273	**0.582**	0.028	-0.130
My body is stiff	0.217	**0.575**	-0.024	-0.032
I am physically tired	0.022	**0.539**	0.098	0.114
My brain is foggy	0.224	**0.485**	-0.059	0.200
***Irritability*** *(ω = 0.884)*				
I am irritated	-0.046	0.033	**0.892**	0.005
I am somewhat annoyed	0.075	-0.009	**0.813**	0.000
I am irascible	-0.054	0.012	**0.806**	0.027
***Lethargy*** *(ω = 0.860)*				
I am not motivated	-0.033	0.047	-0.057	**0.914**
It is hard to do my best	0.069	-0.029	-0.009	**0.796**
I lack concentration	-0.039	-0.033	0.118	**0.723**

The eigenvalues of factors 1, 2, 3, and 4 were 8.904, 2.809, 1.180, and 1.050, respectively, and the extracted factors explained 59.15 of the variance. The bold font indicates that the factor loading of the relevant item was above 0.40

**Table 3:** Factor analysis of subjective health complaints in girls

	Factor loading			
	Factor 1	Factor 2	Factor 3	Factor 4
***Physical and mental pain*** *(ω = 0.906)*				
I have a stomach-ache	**0.893**	0.022	-0.022	-0.082
I feel nauseous	**0.826**	-0.036	-0.071	-0.033
I have diarrhoea	**0.792**	0.029	-0.125	-0.019
I have abdominal pain	**0.783**	-0.117	-0.001	0.030
I feel dizzy	**0.694**	0.215	-0.015	-0.085
I have a headache	**0.660**	0.038	0.029	0.031
I have heart palpitations	**0.658**	-0.027	0.052	0.036
I feel depressed	**0.599**	0.014	0.142	0.095
I feel like crying	**0.470**	0.015	0.225	0.119
***Fatigue*** *(ω = 0.833)*				
I have a stiff neck	-0.081	**0.895**	-0.001	-0.059
I have a backache	0.155	**0.715**	-0.036	-0.046
My body is stiff	0.153	**0.680**	-0.102	0.053
I have eyestrain	-0.049	**0.555**	0.124	0.065
I am physically tired	0.154	**0.425**	0.105	0.095
***Irritability*** *(ω = 0.885)*				
I am irritated	-0.029	0.005	**0.912**	-0.026
I am somewhat annoyed	0.010	0.055	**0.831**	-0.032
I am irascible	-0.022	-0.043	**0.830**	0.020
***Lethargy*** *(ω = 0.858)*				
I am not motivated	0.066	-0.036	-0.073	**0.865**
It is hard to do my best	-0.081	0.088	-0.020	**0.863**
I lack concentration	-0.005	-0.047	0.076	**0.743**

The eigenvalues of factors 1, 2, 3, and 4 were 8.406, 2.681, 1.237, and 1.144, respectively, and the extracted factors explained 59.97 of the variance. The bold font indicates that the factor loading of the relevant item was above 0.40

Based on the results of the factor analysis, we examined differences in the proportions of each symptom during school closure and one year after school reopening using a chi-squared test. Regardless of sex, children were more likely to have irritability- and lethargy-related symptoms during school closure, and a greater proportion felt physical fatigue after school reopened (Tables 4 and 5). Girls were more likely to feel like they were crying during school closure, felt nauseous, and had headaches after the school reopened. Conversely, no differences occurred in the proportions of symptoms related to physical and mental pain in boys.

**Table 4:** Chi-squared test on subjective health complaints during school closure/one year after school reopening in boys

	School closure, *n* (%)		School reopening, *n* (%)		
	Agree	Disagree	Agree	Disagree	*P*
***Physical and mental pain***					
I have a stomach-ache	28 (3.7)	738 (96.3)	23 (3.9)	560 (96.1)	0.782
I have abdominal pain	88 (11.5)	678 (88.5)	65 (11.1)	518 (88.9)	0.846
I have diarrhoea	42 (5.5)	724 (94.5)	36 (6.2)	547 (93.8)	0.590
I have heart palpitations	55 (7.2)	711 (92.8)	41 (7.0)	542 (93.0)	0.917
I feel nauseous	19 (2.5)	747 (97.5)	16 (2.7)	567 (97.3)	0.763
I feel dizzy	26 (3.4)	740 (96.6)	22 (3.8)	561 (96.2)	0.709
I have a headache	65 (8.5)	701 (91.5)	52 (8.9)	531 (91.1)	0.779
I feel depressed	61 (8.0)	705 (92.0)	45 (7.7)	538 (92.3)	0.869
I feel like crying	81 (10.6)	685 (89.4)	54 (9.3)	529 (90.7)	0.426
***Fatigue***					
I have a stiff neck	123 (16.1)	643 (83.9)	97 (16.6)	486 (83.4)	0.775
I have eyestrain	187 (24.4)	579 (75.6)	131 (22.5)	452 (77.5)	0.405
I have a backache	62 (8.1)	704 (91.9)	45 (7.7)	538 (92.3)	0.801
My body is stiff	80 (10.4)	686 (89.6)	49 (8.4%)	534 (91.6)	0.207
I am physically tired	151 (19.7)	615 (80.3)	145 (24.9)	438 (75.1)	**0.023**
My brain is foggy	99 (12.9)	667 (87.1)	78 (13.4)	505 (86.6)	0.806
***Irritability***					
I am irritated	307 (40.1)	459 (59.9)	159 (27.3)	424 (72.7)	**< 0.001**
I am somewhat annoyed	221 (28.9)	545 (71.1)	132 (22.6)	451 (77.4)	**0.010**
I am irascible	291 (38.0)	475 (62.0)	169 (29.0)	414 (71.0)	**0.001**
***Lethargy***					
I am not motivated	367 (47.9)	399 (52.1)	181 (31.0)	402 (69.0)	**< 0.001**
It is hard to do my best	310 (40.5)	456 (59.5)	156 (26.8)	427 (73.2)	**< 0.001**
I lack concentration	413 (53.9)	353 (46.1)	212 (36.4)	371 (63.6)	**< 0.001**

Bold font indicates statistical significance

**Table 5:** Chi-squared test on subjective health complaints during school closure/one year after school reopening in girls

	School closure, *n* (%)		School reopening, *n* (%)		
	Agree	Disagree	Agree	Disagree	*P*
***Physical and mental pain***					
I have a stomach-ache	27 (3.5)	742 (96.5)	27 (5.0)	515 (95.0)	0.187
I feel nauseous	6 (0.8)	763 (99.2)	21 (3.9)	521 (96.1)	**< 0.001**
I have diarrhoea	23 (3.0)	746 (97.0)	26 (4.8)	516 (95.2)	0.090
I have abdominal pain	80 (10.4)	689 (89.6)	73 (13.5)	469 (86.5)	0.089
I feel dizzy	22 (2.9)	747 (97.1)	20 (3.7)	522 (96.3)	0.401
I have a headache	67 (8.7)	702 (91.3)	70 (12.9)	472 (87.1)	**0.014**
I have heart palpitations	58 (7.5)	711 (92.5)	40 (7.4)	502 (92.6)	0.912
I feel depressed	77 (10.0)	692 (90.0)	40 (7.4)	502 (92.6)	0.100
I feel like crying	104 (13.5)	665 (86.5)	50 (9.2)	492 (90.8)	**0.017**
***Fatigue***					
I have a stiff neck	116 (15.1)	653 (84.9)	73 (13.5)	469 (86.5)	0.412
I have a backache	53 (6.9)	716 (93.1)	47 (8.7)	495 (91.3)	0.232
My body is stiff	61 (7.9)	708 (92.1)	34 (6.3)	508 (93.7)	0.254
I have eyestrain	178 (23.1)	591 (76.9)	106 (19.6)	436 (80.4)	0.120
I am physically tired	138 (17.9)	631 (82.1)	137 (25.3)	405 (74.7)	**0.001**
***Irritability***					
I am irritated	308 (40.1)	461 (59.9)	144 (26.6)	398 (73.4)	**< 0.001**
I am somewhat annoyed	257 (33.4)	512 (66.6)	115 (21.2)	427 (78.8)	**< 0.001**
I am irascible	312 (40.6)	457 (59.4)	151 (27.9)	391 (72.1)	**< 0.001**
***Lethargy***					
I am not motivated	346 (45.0)	423 (55.0)	158 (29.2)	384 (70.8)	**< 0.001**
It is hard to do my best	275 (35.8)	494 (64.2)	113 (20.8)	429 (79.2)	**< 0.001**
I lack concentration	404 (52.5)	365 (47.5)	173 (31.9)	369 (68.1)	**< 0.001**

Bold font indicates statistical significance

Table 6 presents the association between adherence to the 24-hour movement behaviour recommendations and subjective health complaints. Girls were less likely to have subjective health complaints on the second factor ‘fatigue’ [OR = 0.845, 95% CI = (0.720–0.992)]. As for grades, 3rd, 4th, 5th, and 6th grades were associated with being at higher risk for the symptoms related to fatigue [OR = 1.646, 95% CI = (1.231–2.201); OR = 1.830, 95% CI = (1.375–2.435); OR = 1.971, 95% CI = (1.484–2.617); OR = 2.423, 95% CI = (1.795–3.270)], and 3rd grade was also associated with increased risk for the symptoms related to the fourth factor ‘lethargy’ [OR = 1.342, 95% CI = (1.014–1.776)]. Subjective health complaints related to the third factor ‘irritability’ and lethargy were less severe one year after school reopening than those during school closure [OR = 0.637, 95% CI = (0.540–0.750); OR = 0.469, 95% CI = (0.397–0.553)]. Adherence to only physical activity recommendations was a factor that decreased the risk of subjective health complaints of fatigue, irritability, and lethargy [OR = 0.416, 95% CI = (0.239–0.727); OR = 0.354, 95% CI = (0.200–0.628); OR = 0.475, 95% CI = (0.279–0.811)]. Children who adhered to only screen time recommendations were less likely to have symptoms related to the first factor (‘physical and mental pain’), irritability, and lethargy [OR = 0.561, 95% CI = (0.353–0.890); OR = 0.594, 95% CI = (0.401–0.881); OR = 0.496, 95% CI = (0.334–0.737)]. Those adhering to two or all three recommendations, except for physical activity and screen time, were more likely to be at low risk for symptoms related to all four factors: physical and mental pain, fatigue, irritability, and lethargy (Table 6).

**Table 6:** Logistic regression analysis on the association of adherence to 24-hour movement behaviour guideline to subjective health complaints

	Factor 1		Factor 2		Factor 3		Factor 4	
	OR	95% CI	OR	95% CI	OR	95% CI	OR	95% CI
Sex								
Boys	Reference		Reference		Reference		Reference	
Girls	1.100	0.926–1.308	**0.845**	**0.720–0.992**	1.025	0.877–1.198	0.908	0.774–1.065
Grade								
1st	Reference		Reference		Reference		Reference	
2nd	1.216	0.903–1.636	1.301	0.977–1.734	1.019	0.782–1.329	1.190	0.908–1.559
3rd	0.955	0.697–1.308	**1.646**	**1.231–2.201**	1.199	0.913–1.576	**1.342**	**1.014–1.776**
4th	1.167	0.862–1.579	**1.830**	**1.375–2.435**	1.039	0.793–1.363	1.161	0.881–1.531
5th	1.214	0.900–1.637	**1.971**	**1.484–2.617**	0.927	0.708–1.214	1.054	0.801–1.388
6th	1.049	0.759–1.449	**2.423**	**1.795–3.270**	1.065	0.799–1.419	0.769	0.575–1.030
Period								
School closure	Reference		Reference		Reference		Reference	
One year after school reopening	0.998	0.831–1.197	1.063	0.898–1.259	**0.637**	**0.540–0.750**	**0.469**	**0.397–0.553**
Compliance category								
None	Reference		Reference		Reference		Reference	
Physical activity	0.720	0.397–1.303	**0.416**	**0.239–0.727**	**0.354**	**0.200–0.628**	**0.475**	**0.279–0.811**
Screen time	**0.561**	**0.353–0.890**	0.733	0.496–1.085	**0.594**	**0.401–0.881**	**0.496**	**0.334–0.737**
Sleep	0.906	0.724–1.134	0.813	0.659–1.002	0.843	0.684–1.038	0.818	0.657–1.017
Physical activity and screen time	0.759	0.268–2.146	0.499	0.186–1.342	**0.348**	**0.123–0.985**	**0.282**	**0.104–0.765**
Screen time and sleep	**0.556**	**0.408–0.758**	**0.435**	**0.326–0.581**	**0.481**	**0.365–0.632**	**0.433**	**0.329–0.571**
Physical activity and sleep	**0.632**	**0.419–0.953**	**0.474**	**0.325–0.693**	**0.672**	**0.471–0.959**	**0.443**	**0.309–0.636**
All three	**0.430**	**0.243–0.760**	**0.552**	**0.345–0.882**	**0.417**	**0.263–0.662**	**0.321**	**0.203–0.507**

OR, odds ratio; CI, confidence interval. All three comprise physical activity, screen time, and sleep. Factors 1, 2, 3, and 4 are termed ‘physical and mental pain’, ‘fatigue’, ‘irritability’ and ‘lethargy’, respectively. Bold font indicates statistical significance

## Discussion

This study investigated subjective health complaints and adherence to 24-hour movement recommendations among school-aged children during COVID-19-related school closures and one year after school reopening. Our results indicate that during school closure, children were more inclined to meet only sleep recommendations. However, upon school reopening, a higher proportion failed to meet any recommendation, meeting only screen time recommendations, or meeting both screen time and sleep recommendations. These findings were consistent, irrespective of sex. Additionally, children were prone to experiencing symptoms of irritability and lethargy during school closures. Multiple logistic regression analyses revealed that individuals adhering to two or all three recommendations, excluding physical activity and screen time, had a lower risk of symptoms related to physical and mental pain, fatigue, irritability, and lethargy compared to those who met none of the recommendations.

It is widely acknowledged that a reduction in physical activity [3–5], an increase in sedentary behaviour (including recreational screen time) [3–6], and an increase in sleep duration [5–7] occurred during pandemic-associated restrictions. Previous studies employing 24-hour movement behaviour guidelines have also indicated that the prevalence of meeting these recommendations presented consistent changes from before to during the pandemic [46–49]. In this study, individuals who met only screen time or sleep recommendations exhibited patterns in line with those of previous studies [46–49]; however, a consistent trend in physical activity was exclusively observed among girls. A survey conducted in Tunisia reported that physical activity decreased during the pandemic among school-aged children, with a decrease of 7% in boys and 17% in girls [50]. However, other studies present contrary results, indicating that the decrease in physical activity is smaller in girls than in boys [51, 52]. Our results, showing a greater proportion of girls meeting the physical activity recommendation one year after school reopening, could support the former report and suggest that the negative impact of school closures due to the pandemic on children’s physical activity was more evident in girls than in boys. This difference in sex may be explained by the following reasons: boys may be more active than girls [53, 54], and girls may be more likely to comply with restrictive measures [55]. Additionally, differences in government policies for the prevention of infection spreading, may have contributed to the disparities between the current findings and those of previous studies [51, 52]. In Japan, the government only recommended, but did not enforce, stay-at-home measures. Consequently, the relatively mild restrictions could have permitted boys to engage in voluntary physical activities.

In addition, the impact of the pandemic-associated restrictions on children’s movement behaviours, especially physical activity, seemingly persisted in the aftermath. This was suggested by a Spanish study indicating that adherence to the 24-hour movement behaviour guidelines in children was significantly lower after the pandemic than before it [56].

We hypothesised that attending school would have a positive role in children’s daily movement behaviours; nevertheless, our results revealed an increase in the number of individuals who did not meet any of these recommendations one year after the school reopened. One possible explanation for this outcome could be the increase in individuals who could not meet even the sleep recommendations after schools reopen. Short sleep duration among Japanese children remains a major concern [57]. This issue is so serious that the United Nations Committee on the Rights of the Child has recommended strengthening efforts to ensure every child’s right to sufficient rest and leisure [58]. Our results could reflect the fact that that since the schools reopened, the numerous activities that children are involved in contribute to their short sleep durations. Indeed, a previous study in Japan observed a lack of free time among school-aged children [59], which could explain why they were unable to get sufficient sleep.

Several reports have suggested that there is a high risk of poor concentration, inattention, and irritability in children during pandemics [60–62]. These findings are consistent with the current results. Further, South Korean and Canadian surveys have indicated that children’s loneliness due to limited social interaction or social isolation during the pandemic impacted their mental health, including depression, irritability, and attention issues [63, 64]. Taken together, the increase in irritability and lethargy observed in this study could be associated with a reduction in social interactions due to school closures. Since social interactions play an important role in children’s psychological development [65], attention should be paid to the current and future mental health of children experiencing extreme social isolation.

Girls were more likely to feel like crying during school closure and experience nausea and headaches one year after school reopening. Academic pressure has a stronger effect on psychosomatic symptoms in girls than in boys [66, 67]. Additionally, school closures contribute to reducing academic pressure on children [68–70]. Thus, our finding of an increase in subjective health complaints, such as nausea or headaches, after the school reopening in girls could be explained by school-related stress.

One key finding is that adhering to two movement behaviour guidelines, particularly combining screen time and sleep or physical activity and sleep, along with meeting all three guidelines, reduces the risk of subjective health complaints among school-aged children. This aligns with previous research suggesting that meeting multiple recommendations improves mental health outcomes in children and adolescents, including depression [27, 29–31], anxiety [27, 29–31], quality of life [33], self-rated physical and mental health [34], and internalising and externalising behaviours [32]. Notably, meeting at least two recommendations, including sleep, is essential for decreasing the risk of subjective health complaints. This is a remarkable result for Japanese children, as the proportion of children who do not get enough sleep is the highest in the world. Several reports have suggested that sufficient sleep has a stronger impact on children’s mental health than physical activity or sedentary behaviour [29, 71]. These reports and our findings highlight the significance of adhering to sleep recommendations and imply that ensuring sufficient sleep should be a priority for better mental health among school-aged children. If they cannot meet all three recommendations, meeting an additional recommendation in addition to the sleep recommendation could be effective in preventing mental health issues. In this study, we did not separately analyse 24-hour movement behaviours and subjective health complaints during school closures and reopening. Results of previous research have not been entirely consistent regarding the association between 24-hour movement behaviours and mental health indicators, even when similar indicators, such as depression and anxiety, are used [31, 72]. Thus, the association may vary depending on survey periods or sample characteristics (e.g. age and regions) or mental health assessment tools. Nevertheless, strategies that consider the priority of each movement behaviour are necessary, even in abnormal situations, such as social restrictions due to the pandemic.

Conversely, meeting sleep recommendations alone did not significantly reduce the risk of subjective health complaints in this study. One reason for this discrepancy could be that sleep timing was not considered. A later timing of sleep is associated with more headaches, stomach-aches, and backaches among school-aged children in Canada [73]. Not only this study but also other studies have suggested an association between the timing of sleep and mental health among children [74, 75]. Sleep patterns could have substantially differed between the two periods: during school closure and one year after school reopening. Studies on children’s sleep patterns during lockdowns and school closures indicate a tendency for many children to go to bed and wake up later [7, 76]. Although previous studies have demonstrated the independent effect of physical activity, screen time, and sleep on subjective health complaints in school-aged children [19, 25, 26], our study has shown that meeting only one recommendation (physical activity, screen time, or sleep) did not reduce the risk of subjective health complaints. This suggests that movement behaviours interact with each other, and a negative effect caused by not meeting one behaviour offsets a positive effect gained by meeting the other; therefore, examining the association between the integrated 24-hour movement behaviour guidelines and mental health outcomes is crucial and needed for an effective approach. Taken together, adherence to the 24-hour movement behaviour guidelines is a useful indicator for promoting healthy movement behaviours to improve mental health among children from a public health perspective. However, it is essential to accumulate more evidence to explore the association between combinations of movement behaviours and subjective health complaints. This analysis may need to consider the timing at which each recommendation was achieved.

Previous studies have revealed the prevalence of subjective health complaints in girls [19, 21, 22, 77] and older children [19, 21, 22]. This study examined the differences in sex and age only for fatigue-related symptoms. Additionally, girls were at a lower risk of experiencing these symptoms than boys, which is inconsistent with prior findings [19, 21, 22]. One reason for this difference could be the dissimilar grade ranges of participants. Participants included all elementary school grades. Tanaka and colleagues reported that the scores of subjective fatigue symptoms among boys were higher than those among girls in the third and fourth grades in Japan [41]. Therefore, sex differences could exhibit opposite trends with age. Moreover, a Japanese study on subjective fatigue symptoms revealed that scores for fatigue symptoms were significantly greater in the fifth and sixth grades than in the third and fourth grades [41]. Our results are consistent with this finding and suggest the necessity of a preventive approach for subjective health complaints, particularly those with fatigue symptoms, in the population before early adolescence.

### Strengths and limitations

To our knowledge, this study is the first to analyse of subjective health complaints among school-aged children during school closures due to the pandemic and one year after schools reopened. While investigations into other mental health outcomes during school closures and surrounding periods exist, they primarily focused on depression and anxiety. This study yields key findings, offering insights into children’s mental health concerns during unprecedented and massive disasters or crises, such as the COVID-19 pandemic.

However, this study had some limitations. First, it employed a cross-sectional design, and participants were recruited using snowball sampling. While participants in this study were drawn from the same schools in both surveys, this was insufficient to completely exclude bias and establish cause-and-effect relationships. Fortunately, we were able to obtain the data twice: in an irregular situation, during school closure, and one year after school reopening. However, future studies on changes in both movement behaviours and subjective health complaints in specific circumstances and settings should include longitudinal data to provide stronger evidence. Second, the data on movement behaviours among the children were self-reported. The reliability and validity of screen time and sleep measures have not yet been confirmed. These should be examined in future studies, although some Japanese national surveys have used similar questions. Further, for a comprehensive observation of movement behaviours and a thorough examination of the genuine relationship between combinations of movement behaviours and subjective health complaints, it is essential to conduct further examinations using accelerometers. Third, this study included a limited number of confounding factors: sex and grade. Sex and gender differences are key factors influencing subjective health complaints [22, 77]. However, further analysis is required to assess various factors and determine what factors potentially affect subjective health complaints among school-aged children. For instance, socioeconomic status is associated with mental health outcomes among children and adolescents [78].

## Conclusions

This study found that school-aged children, regardless of sex, were more likely to meet ‘only sleep’ recommendations and experience symptoms related to irritability and lethargy during COVID-19 pandemic-related school closures as compared to one year after school reopening. It also demonstrated that individuals meeting two or all three recommendations (excluding physical activity and screen time) had a lower risk of symptoms related to physical and mental pain, fatigue, irritability, and lethargy as compared to those who met none of the recommendations. These findings underscore the significance of school-aged children meeting either the physical activity or screen time recommendations, in addition to fulfilling sleep recommendations, for their subjective health.

## Data Availability

The datasets used and/or analysed during this study are available from the corresponding author on reasonable request.

## Abbreviations

CI: confidence interval
COVID-19: coronavirus disease 2019
OR: odds ratio
